# Clinical and immunological characterization of *NFKB1* haploinsufficiency in Japan

**DOI:** 10.3389/fimmu.2026.1866459

**Published:** 2026-07-08

**Authors:** Kunihiko Moriya, Yuji Kamiyama, Ryo Ogino, Shuya Kaneko, Takeshi Isoda, Takahiro Kamiya, Yuki Sakai, Fumi Hirose, Hidetoshi Hagiwara, Itaru Iwama, Ryusuke Nambu, Yoji Uejima, Fumiaki Sakura, Miyuki Tsumura, Kazushi Izawa, Yusuke Matsuda, Naoko Nakatani, Akihiro Tamura, Tomo Nozawa, Masaki Shimizu, Taizo Wada, Satoshi Okada, Hirokazu Kanegane, Kohsuke Imai

**Affiliations:** 1 Department of Pediatrics, National Defense Medical College, Saitama, Japan; 2 Department of Antibody Drug Development, Tohoku University Graduate School of Medicine, Sendai, Japan; 3 Department of Pediatrics, Graduate School of Medicine, Yokohama City University, Yokohama, Japan; 4 Department of Pediatrics, Kyoto University Graduate School of Medicine, Kyoto, Japan; 5 Department of Pediatrics and Developmental Biology, Graduate School of Medical and Dental Sciences, Institute of Science Tokyo, Tokyo, Japan; 6 Division of Gastroenterology and Hepatology, Saitama Children’s Medical Center, Saitama, Japan; 7 Department of Infectious Diseases and Immunology, Division of Infectious Diseases and Immunology, Saitama Children’s Medical Center, Saitama, Japan; 8 Department of Pediatrics, Hiroshima University Graduate School of Biomedical and Health Sciences, Hiroshima, Japan; 9 Department of Pediatrics, School of Medicine, Institute of Medical, Pharmaceutical and Health Sciences, Kanazawa University, Kanazawa, Japan; 10 Department of Pediatrics, Kobe University Graduate School of Medicine, Kobe, Japan; 11 Department of Child Health and Development, Graduate School of Medical and Dental Sciences, Institute of Science Tokyo, Tokyo, Japan

**Keywords:** autoimmunity, autoinfammatory disease, common variable immunodeficiency (CVID), inborn errors of immunity (IEI), NFKB1

## Abstract

**Background:**

*NFKB1* haploinsufficiency caused by monoallelic loss-of-function variants in *NFKB1* results in a CVID-like phenotype or other forms of hypogammaglobulinemia.

**Objective:**

This study aims to characterize the clinical and immunological profiles of 21 individuals from nine families with this disorder.

**Methods:**

Gene panel sequence and/or whole exome sequence, followed by Sanger sequencing, were used to identify and confirm *NFKB1* variants. Immunophenotyping were performed by flow cytometry. The type I interferon signature was determined by quantitative PCR.

**Results:**

Of the nine *NFKB1* variants, seven novel heterozygous variants were identified in this study. Ten individuals were clinically asymptomatic, while 11 were symptomatic, resulting in clinical penetrance of 52%. However, immunologic abnormalities were observed in all asymptomatic family members tested (n=9). The main presenting symptoms in symptomatic individuals were respiratory tract infections (7/11) and autoimmune or autoinflammatory features (7/11). Interestingly, two patients had Moyamoya disease, and one patient was complicated by alopecia and rheumatoid arthritis. Immunological analyses were performed in 19 individuals, including 9 asymptomatic, and revealed that 58% had low absolute T cell counts and that 72% of individuals displayed inverted CD4/CD8 T-cell ratio. B-cell lymphopenia and decreased switch memory B cells were observed in 42% and 61% of individuals, respectively. A type I interferon signature was not observed.

**Conclusions:**

In addition to hypogammaglobulinemia/CVID-like phenotype, *NFKB1* variants have been associated with a wide range of manifestations, ranging from various organ involvements to autoimmunity. Autoinflammatory features have also been reported; however, the presence of type I interferon signatures was not specifically addressed in this study.

## Introduction

Inborn errors of immunity (IEI) are immune abnormalities caused by gene variants that confer susceptibility to various infections, autoinflammatory disorders, autoimmunity, and malignancies. More than 500 genetic disorders have been recognized (1). Most human IEIs affecting the canonical NF-κB pathway impair both innate and adaptive immunity (2). Some clinical manifestations of these disorders are unrelated to hematopoiesis, including anhidrotic ectodermal dysplasia, lymphedema, and osteopetrosis (3). Studies of NF-κB-related disorders have improved our understanding of the clinical and immunological phenotypes observed in patients with inborn errors affecting the core components of the canonical NF-κB pathway.

*NFKB1* encodes the ubiquitous nuclear transcription factor κB subunit NF-κB1, which is synthesized as the p105 precursor protein and processed to generate the p50 subunit. The p50 subunit forms heterodimers with RelA and c-Rel, which are retained in the cytoplasm by IκB inhibitors. Upon stimulation by external signals, such as pro-inflammatory cytokines or pathogens, the IκB kinase (IKK) complex is activated and phosphorylates IκB, leading to its ubiquitination and proteasomal degradation. This process releases NF-κB dimers, allowing them to translocate into the nucleus, where they bind to specific DNA regions and initiate transcription of target genes involved in immune and inflammatory responses (4).

NF-κB1 plays a pivotal role in the canonical NF-κB pathway, a crucial signaling mechanism in immune responses, inflammation, and cell survival (4). Hypogammaglobulinemia and common variable immunodeficiency (CVID)-like phenotypes have been associated with *NFKB1* variants (5). Among Europeans, loss-of-function variants in *NFKB1* have been reported as the most common monogenic cause for CVID, accounting for approximately 4% of cases (6).

In addition to CVID-like phenotype or other forms of hypogammaglobulinemia, *NFKB1* variants have been associated with a broad spectrum of clinical manifestations, ranging from multi-organ involvements to autoimmunity (7–9). Autoinflammatory features have also been reported in this disorder. In the present study, we conducted a multicenter survey of individuals with monoallelic *NFKB1* variants in Japan. We further evaluated type I interferon signatures to investigate the mechanisms underlying the autoinflammatory manifestations associated with this condition.

## Materials and methods

### Patients/cohort

Our cohort consisted of 21 consenting individuals in nine different families, carrying nine distinct heterozygous *NFKB1* pathogenic variants. All consenting, potential living carriers in these families were systematically interviewed, screened genetically, and their available patient records studied.

### Blood collection

Blood samples were collected from patients and relatives after written informed consent had been obtained. Blood samples were drawn as part of the diagnostic procedure. This study was conducted in accordance with the recommendations of the Declaration of Helsinki and was approved by the Ethics Committee of National Defense Medical College (No.4738).

### Sequencing

We identified variants using by whole-exome sequencing (WES), or a gene panel containing 485 primary immunodeficiency-associated genes listed in IUIS2022 (10). All variants were confirmed by Sanger sequencing performed as previously described (11).

### Whole-exome sequencing

Genomic DNA (3 µg) extracted from the peripheral blood cells of the patient was sheared with a Covaris S2 Ultrasonicator (Covaris). An adaptor-ligated library was prepared with the Paired-End Sample Prep kit V1 (Illumina). Exome capture was performed with the Sure Select Human All Exon kit (Agilent Technologies). Single-end sequencing was performed on an Illumina Genome Analyzer IIx (Illumina), generating 72-base reads.

### Type I interferon signature analysis in whole-blood leukocytes

The type I interferon signature was determined as previously described (12, 13). Total RNA was extracted from whole blood collected into PAXgene Blood RNA tubes (762165, PreAnalytiX, Hambrechtikon, Switzerland) and reverse transcribed by a PrimeScript II 1^st^ strand cDNA synthesis kit (6210A, Takara Bio, Shiga, Japan). Quantitative PCR was performed with TaqMan Gene Expression Master Mix (4369016, Applied Biosystems) with probes (IFI27, Hs01086370_m1; IFIT1, Hs00356631_g1; RSAD2, Hs01057264_m1; SIGLEC1, Hs00988063_m1; ISG15, Hs00192713_m1; IFI44L, Hs00199115_m1; BACT, Hs01060665_g1, Applied Biosystems) on a StepOnePlus Real-Time PCR system (Thermo Fisher Scientific, Waltham, MA, US). The expression levels of each transcript were determined in triplicate and normalized to the level of β-actin. The results are shown relative to a single calibrator. The median relative quantification (RQ) value of the six IFN-stimulated genes (ISGs) was used to calculate the “IFN score” for each patient. IFN scores greater than +2 SD of the average of 11 healthy controls (5.04) were designated as positive. For each autosomal dominant (AD) *NFKB1* patient, multiple blood draws were taken at regular follow-up appointments in the absence of obvious signs of infection or fever. For disease controls, data from 3 Aicardi–Goutières syndrome (AGS) patients (2 genetically diagnosed: IFIH1 (p.R779H/WT) and IFIH1 (p.R720Q/WT), and one clinically diagnosed) was used. Two independent experiments were performed to confirm the results.

### Flow cytometry

Human peripheral blood mononuclear cells were isolated by Ficoll–Hypaque density centrifugation (Amersham Pharmacia Biotech, Sweden) from whole blood and cultured in RPMI medium supplemented with 10% fetal bovine serum (Gibco BRL, Invitrogen, USA). The PBMCs were washed twice and resuspended in phosphate-buffered saline. Next, the PBMCs (1, 000, 000) were stained with cocktails of monoclonal antibodies as previously reported (14). For data acquisition, a BD LSR Fortessa flow cytometer (Becton Dickinson, Franklin Lakes, NJ) was used. The samples and data were analyzed with FlowJo software (FlowJo LLC, Ashland, OR).

### Statistical analysis

**Figure 1:**
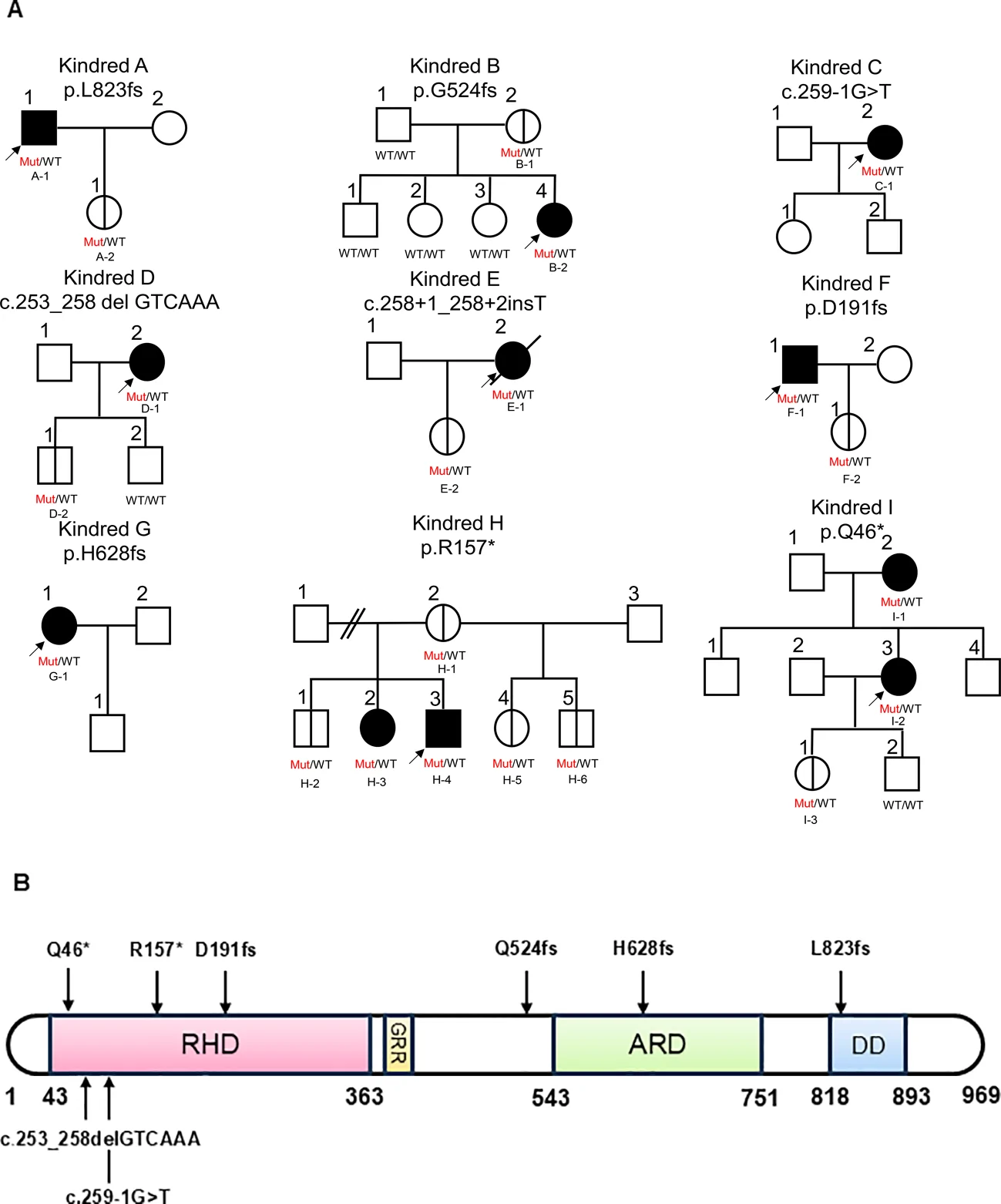
AD *NFKB1* deficiency in 9 families. **(A)** Pedigree of the 9 unrelated families showing familial segregation of the different *NFKB1* alleles. Male and female individuals are represented by squares and circles, respectively. Symptomatic individuals are represented by closed black symbols, and asymptomatic carriers are indicated by a black vertical line. **(B)** Schematic representation of the *NFKB1* gene. The positions of the variants observed in the individuals are indicated by arrows. RHD, Rel homology domain; GRR, glycine-rich region, ARD, ankyrin repeat domain; DD, death domain.

Statistical analyses were performed using R software, version 4.3.1 (R Project for statistical computing). Age-dependent cumulative incidence of clinical manifestations among variant carriers was estimated using the Kaplan–Meier method. Symptomatic carriers were treated as events at the age of symptom onset, whereas asymptomatic carriers were censored at the age at last clinical evaluation. Cumulative incidence and 95% confidence intervals were calculated from Kaplan–Meier estimates. Results for **Figure 1** were analyzed using a one-way ANOVA with a Dunnett’s multiple comparisons test. All statistical analyses, described above, were performed using the GraphPad Prism software version 8.00 (GraphPad Software, La Jolla, California, USA, www.graphpad.com).

## Results

### Patients/cohort

**Figure 2:**
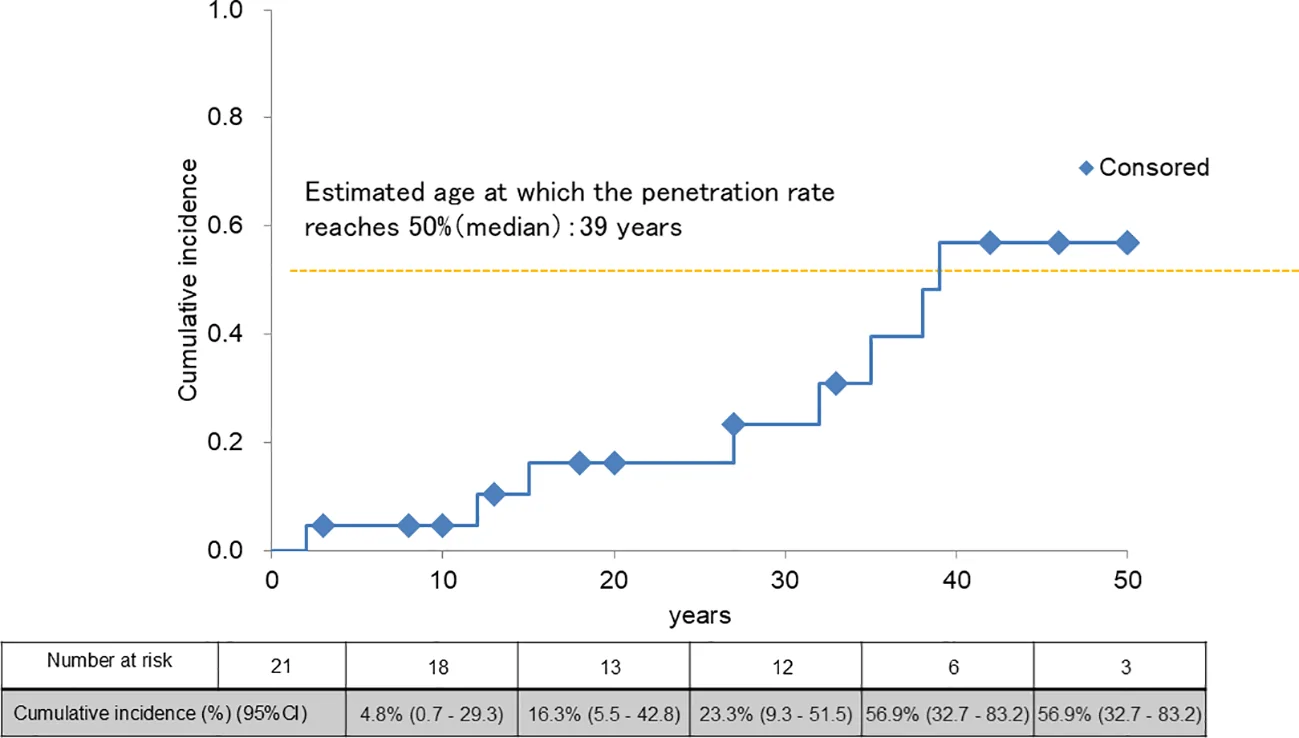
Cumulative incidence up to age 50.

We studied 21 individuals from nine unrelated kindred with monoallelic *NFKB1* variants (**Figure 1A**). These variants were predicted to cause *NFKB1* haploinsufficiency due to nonsense or frameshift variants. and splice-site variants (**Table 1**). Except P8, symptomatic individuals were alive at time of reporting. Study subjects carried nine distinct heterozygous *NFKB1* variants: c.2465dupA: (p.Leu823fs), c.1569_1570delAG: (p.G524fs), c.259-1G>T, c.253_258 del GTCAAA, c.258 + 1_258 + 2insT, c.572dupA (p.Asp191fs), c.1883dupA: (p.His628fs), c.469C>T: (p.R157*), c.136C>T: p.Q46* (15) (**Figure 1B**). The pathogenicity for the previously reported variants, p.R157* has been shown in *in vitro* experiments (16). Other variants demonstrated minor allele frequency (MAF) was 0.00, and no past records were represented at gnomAD v4.1.1, TMM 61KJPN in the Japanese cohort (The 61KJPN dataset from jMORP (https://jmorp.megabank.tohoku.ac.jp/) and were not reported in the Human Gene Mutation Database (https://www.hgvd.genome.med.kyoto-u.ac.jp/) (**Table 1**). The variants except c.258 + 1_258 + 2insT were classified as likely pathogenic according to the Flanklin American College of Medical Genetics (https://franklin.genoox.com/) because the loss of NF-κB1 function is known to cause CVID, and the eight variants identified were null. As for c.258 + 1_258 + 2insT, showed a highly predicted to effect splicing by in silico analysis using Splice AI software (https://spliceailookup.broadinstitute.org/#variant=X-101359619%20C-A&hg=38&distance=500&mask=1) (**Table 1**). Of the studied 21 *NFKB1* variant carriers, 15 (71%) were women and 6 (29%) men. In this family-based cohort, the estimated cumulative risk of symptom manifestation reached 52%. Eleven of the 21 were symptomatic, suggesting a clinical penetrance of at least 52%. The estimated age at which the penetration rate reaches 50% was 39 years (**Figure 2**).

**Table 1:** List of MAF and ACMG classification of the variants.

Kindred	Coding change	Amino acid change	Variant type	Novel variant	gnomAD	TMM 61KJPN	ACMG/AMP	SpliceAI
A	NC_000004.12:g.102612477 T>TA NM_003998.4:c.2465dupA	p.L823fs	frameshift	Novel	0	0	Likely Pathogenic PVS1, PM2	
B	NC_000004.12:g.102597592 CAG>C NM_003998.4:c.1569_1570delAG	p.G524fs	frameshift	Novel	0	0	Likely Pathogenic PVS1, PM2	
C	NC_000004.12:g.102566986G>T NM_003998.4:c.259-1G>T		essential splice site	Novel	0	0	Likely Pathogenic PVS1, PM2	
D	NC_000004.12:g.102537950 GGTCAAA>G NM_003998.4:c.253_258 del GTCAAA		frameshift	Novel	0	0	Likely Pathogenic PVS1, PM2	
E	NC_000004.12:g.102537957 G>GT NM_003998.4:c.258+1_258+2insT			Novel	0	0	VUS PM2, PP3	Splice-Altering / strong (0.82)
F	NC_000004.12:g.102578880 G>GA NM_003998.4:c.572dupA	p.Asp191fs	frameshift	Novel	0	0	Likely Pathogenic PVS1, PM2	
G	NC_000004.12:g.102606625 C>CA NM_003998.4:c.1883dupA	p.His628fs	frameshift	Novel	0	0	Likely Pathogenic PVS1, PM2	
H	NC_000004.12:g.102576937 C>T NM_003998.4:c.469C>T	p.R157*	nonsense	Reported	0	0	Pathogenic PVS1, PM2,PS4, PP5	
I	NC_000004.12:g.102533862 C>T NM_003998.4: c.136C >T	p.Q46*	nonsense	Reported	0	0	Likely Pathogenic PVS1, PM2, PP1	

### Clinical manifestations

**Figure 3:**
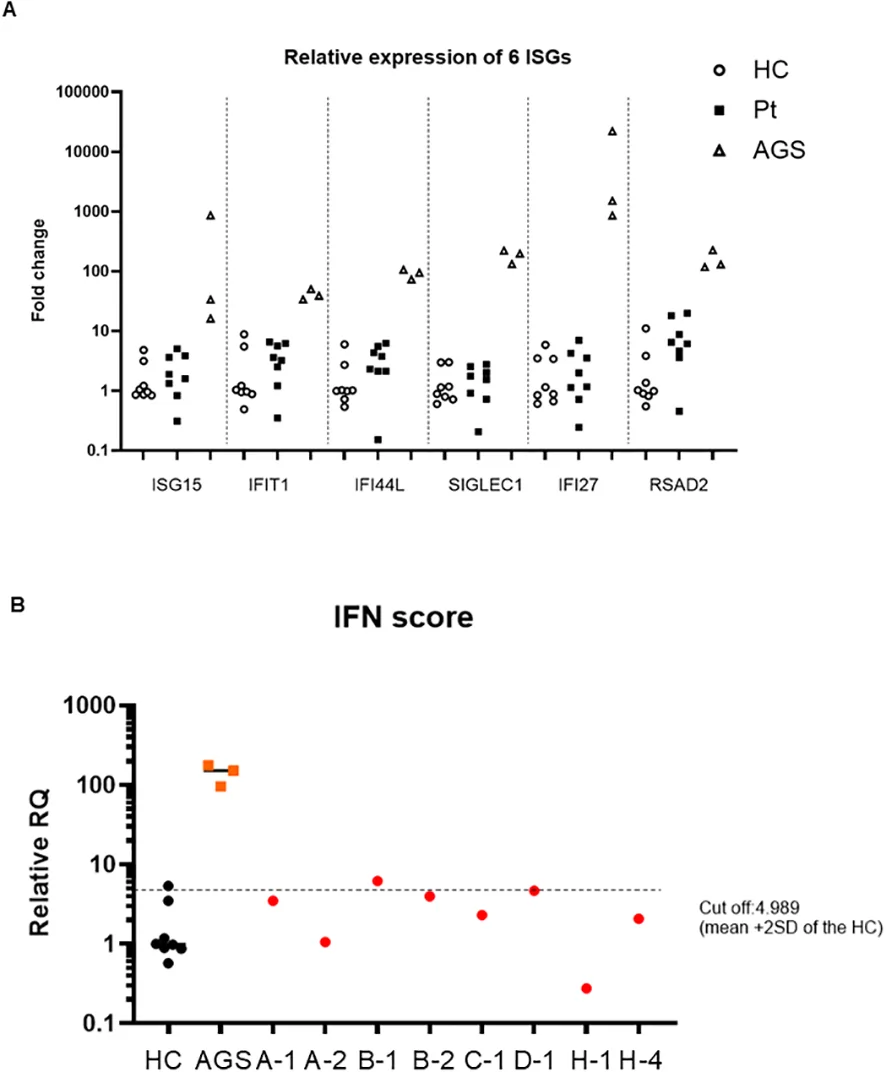
AD *NFKB1* variants do not underlie type I interferonopathy. **(A)** Relative expression of six ISGs and **(B)** IFN scores of 11 healthy controls, 8 patients with AD *NFKB1* variants, and 3 patients with Aicardi–Goutières syndrome (AGS) were evaluated by qRT–PCR. The relative abundance of each transcript was normalized to the expression level of ß-actin, and the results are shown relative to a single calibrator. The median of the relative quantification of the 6 ISGs was used to calculate the “IFN score” for each patient. IFN scores greater than +2 SD of the average of 11 healthy controls (5.04) were designated as positive. Two independent experiments were performed to confirm the results.

Clinical characteristics of all 21 individuals studied are summarized in **Table 2** and **Table 3A**. The main presenting symptoms among symptomatic individuals were respiratory tract infections (7/11) and autoimmune or autoinflammatory features (7/11). Two patients presented non-clonal lymphoproliferation. Other presenting features included variable gastrointestinal manifestations (4/11) and skin manifestations (5/11). Notably, two individuals (H-3, H-4) presented with Moyamoya disease (MMD) and required surgical intervention before being diagnosed with *NFKB1* haploinsufficiency. Autoinflammatory conditions have been reported in patients with *NFKB1* haploinsufficiency (8, 9), leading us to hypothesize that these patients might exhibit an elevated type I interferon signature. In eight individuals (A-1, A-2, B-1, B-2, C-1, D-1, H-1, H-4) with heterozygous *NFKB1* variants, we evaluated the interferon signature, comparing them with healthy controls as negative controls and with three patients with AGS as positive controls. However, this analysis did not reveal increased type I interferon signatures in the individuals (**Figure 3A, B**).

**Table 2:** Clinical characteristics of individuals with AD NFKB1 deficiency.

Kindred	A		B		C	D				E		
Patient number	A-1	A-2	B-1	B-2	C-1	D-1		D-2		E-1		E-2
Sex	M	F	F	F	F	F		M		F		F
Age at diagnosis	35	asymptomatic	asymptomatic	2	32	52		asymptomatic		55		asymptomatic
Age (y) at Immunological Analysis	42	1	39	9	38	57		NT		58		24
Age (y) at last follow-up	45	3	42	12	41	62		33		58		27
Gene Mutation	c.2465dupA (p.L823fs)	c.2465dupA (p.L823fs)	c.1569_1570delAG (p.G524fs)	c.1569_1570delAG (p.G524fs)	c.259-1G>T	c.253_258delGTCAAA		c.253_258delGTCAAA		c.258+1_258+2insT		c.258+1_258+2insT
Infection Episode (pathogen)	RTI Septic Arthritis of the Hip, Cellulitis, Onychomycosis	No	No	RTI	RTI	RTI		No		Cellulitis, CMV gastric ulcer, Bacteremia, Pneumonia		No
Others	Alopecia			PFAPA Short stature	Warts	Colon Polyp, Hepatic Hemangioma				BA, LPD, Portal hyper tension, NRH		
Treatment	SCIG, ABx	No	No	SCIG	SCIG, ABx	SCIG, ABx		No		SCIG GCV, ABx		No
Outcome	Alive	Alive	Alive	Alive	Alive	Alive		Alive		Death		Alive
Kindred	F		G	H						I		
Patient number	F-1	F-2	G-1	H-1	H-2	H-3	H-4	H-5	H-6	I-1	I-2	I-3
Sex	M	F	F	F	M	F	M	F	M	F	F	F
Age at diagnosis	38	asymptomatic	39	asymptomatic	asymptomatic	15	12	asymptomatic	asymptomatic	54	27	asymptomatic
Age (y) at Immunological Analysis	43	15	39	43	17	15	12	10	7	NT	31	5
Age (y) at last follow-up	46	18	42	46	20	18	15	13	10	60	34	8
Gene Mutation	c.572dupA (p.D191fs)	c.572dupA (p.D191fs) hetero	c.1883dupA(p.H628fs)	c.469C>T (p.R157*)	c.469C>T (p.R157*)	c.469C>T (p.R157*)	c.469C>T (p.R157*)	c.469C>T (p.R157*)	c.469C>T (p.R157*)	c.136C>T (p.Q46*)	c.136C>T (p.Q46*)	c.136C>T (p.Q46*)
Infection Episodes (pathogen)	Cellulitis, Invasive pneumococcal disease	No	RTI	No	No	No	No	No	No	No	Rash, stomatitis	No
Others	LPD					MMD	MMD, ITP, EGID			Rheumatoid arthritis		
Treatment	SCIG, PSL, ABx Sirolimus	No	SCIG, ABx	No	No	SCIG	SCIG, PSL, TNF inhibitor	No	No	PSL	SCIG	IVIG
Outcome	Alive	Alive	Alive	Alive	Alive	Alive	Alive	Alive	Alive	Alive	Alive	Alive

**Table 3:** Clinical and immunological spectrum of individuals with AD NFKB1 deficiency.

(A) Clinical spectrum of individuals with AD NFKB1 deficiency																								
Clinical phenotype	A-1	A-2	B-1	B-2	C-1	D-1	D-2	E-1	E-2	F-1	F-2	G-1	H-1	H-2	H-3	H-4	H-5	H-6	I-1	I-2	I-3			
Respiratory tract infections																						33	%	(7/21)
Non-infections lung complications																						5	%	(1/21)
Gastrointestinal manifestations																						19	%	(4/21)
Neurodisorders																						10	%	(2/21)
Autoimmue disorders																						33	%	(7/21)
Lymphoproliferation																						10	%	(2/21)
Skin manifestations																						24	%	(5/21)
skin infections																						19	%	(4/21)
Non-infections skin complications																						10	%	(2/21)
(B) Immunological spectrum of individuals with AD NFKB1 deficiency																								
Immunological feature	A-1	A-2	B-1	B-2	C-1	D-1	D-2	E-1	E-2	F-1	F-2	G-1	H-1	H-2	H-3	H-4	H-5	H-6	I-1	I-2	I-3			
Low absolute CD3+ or CD4+ or CD8+ T cells							NT												NT			58	%	(11/19)
Inverted CD4+/CD8+ T cell ratio					NT		NT												NT			72	%	(13/18)
Low naïve CD4+ or CD8+ T cells					NT		NT												NT			39	%	(7/18)
Low absolute CD19+B cells							NT												NT			42	%	(8/19)
Low memory CD19+B cells					NT		NT												NT			61	%	(11/18)
Low IgG			NT				NT					NT	NT				NT	NT	NT			64	%	(9/14)
Low IgA			NT				NT					NT	NT				NT	NT	NT			50	%	(7/14)
Low IgM			NT				NT					NT	NT				NT	NT	NT			42	%	(6/14)

NT, not tested.

### Immunological features

Immunological investigations were performed in 19 individuals with *NFKB1* variants, including nine asymptomatic carriers, and the results are summarized in **Table 3B** and **Supplementary Table 1**. Eleven (58%) of them showed low absolute T-cell counts and 13 (72%) displayed an inverted CD4/CD8 T-cell ratio. Similarly, seven (39%) showed a reduction of naive T cells (CD4 ^+^ or CD8^+^). Double-negative αβ T cells were not increased in all individuals (**Supplementary Table 1**), while B-cell lymphopenia was observed in eight (42%) and decreased switched memory B cells were found in 11 (61%) individuals. In addition, humoral abnormalities were frequent, with low IgG levels (64%), low IgA levels (50%), low IgM levels (42%) by assessing age-adjusted laboratory reference intervals.

We identified ten asymptomatic family members (A-2, B-1, D-2, E-2, F-2, H-1, H-2, H-5, H-6, I-3) carrying the same *NFKB1* pathogenic variants as their symptomatic relatives. Interestingly, some form of immunologic abnormality was observed in all tested asymptomatic family members (**Table 3B**).

### Treatment

Among the 11 symptomatic individuals, antibiotic prophylaxis and immunoglobulin replacement therapy were used in 6 (55%) and 11 (100%) individuals before genetic diagnosis, respectively (**Table 2**). Immunosuppressive drugs, such as prednisolone, sirolimus, and TNF inhibitors were administered to 3 (27%) individuals (F-1, H-4, I-1). No individuals underwent allogeneic hematopoietic stem cell transplantation.

## Discussion

Here, we report the clinical and immunological features of 21 Japanese individuals carrying pathogenic *NFKB1* variants. The clinical penetrance of *NFKB1* variants in our cohort was 52%, consistent with previous reports estimates ranging from 50 to 100% (7–9). The age at which penetrance reached 50% was 39 years, supporting age-dependent disease expression. We observed substantial intrafamilial heterogeneity, even among members of the same family, in line with incomplete and age-related penetrance. Consistent with this, large international cohort data indicate that penetrance increases with age and may approach 100% in individuals aged ≥60 years (8). Importantly, obligate carriers may remain asymptomatic despite significant hypogammaglobulinemia, highlighting the variable expressivity of *NFKB1* haploinsufficiency. Given the age dependency and the potential for severe complications, genetic evaluation of close relatives should be considered, and longitudinal follow-up is warranted even in asymptomatic or subclinically affected carriers. In addition to recurrent respiratory tract infections, autoimmunity and autoinflammation with diverse clinical manifestations predominated in our cohort. The comparatively lower prevalence of CVID/hypogammaglobulinemia than in previous reports likely reflect differences in study design and patient selection. As earlier studies primarily screened cohorts enriched for hypogammaglobulinemia or CVID, *NFKB1* deficiency may be underrecognized in patients who present mainly with autoimmune or autoinflammatory phenotypes.

Type I interferonopathies are monogenic disorders characterized by constitutive upregulation of type I IFN production and are typically associated with developmental abnormalities, immunodeficiency, and autoinflammation (17). We previously reported six patients with *RELA* dominant-negative variants who exhibited autoinflammatory and autoimmune manifestations accompanied by elevated expression of multiple ISGs, consistent with a type I IFN signatures (13). Mechanistically, RelA (p65) forms heterodimers with the cleaved product of NF-κB1 (p50), and the p65:p50 complex serves as a canonical driver of proinflammatory gene transcription (4). In addition, a study using the THP-1 monocyte cell line suggested that the p.R157* variant, identified in Kindred H, is associated with dysregulated type I IFN signaling and enhanced NLRP3-mediated immune responses (18). Given that *NFKB1* haploinsufficiency can be associated with inflammatory phenotypes, we hypothesized that affected individuals might exhibit an elevated interferon signature. However, no increase in type I IFN signature was detected in the eight individuals analyzed in our cohort (**Figures 3A, B**). This finding is consistent with a previous report of *NFKB1* haploinsufficiency in two adult siblings in whom whole-blood analysis did not show upregulated type I IFN signaling (19). Collectively, these observations suggest that autoinflammation in *NFKB1* haploinsufficiency is unlikely to be primarily driven by type I IFN and may instead arise through mechanisms distinct from those underlying classical type I interferonopathies.

Neurological disorders were observed in 10% of individuals, consistent with the literature, reporting a prevalence of 10-40% across various cohorts (7–9). In our two patients (H-3, H-4), the diagnosis was not prompted solely by the manifestation as noninfectious vasculitis with CNS involvement, nor was CVID initially suspected. Instead, immune dysregulation was suspected due to steroid-dependent gastrointestinal symptoms, and WES subsequently identified previously reported *NFKB1* variants (c.469C>T, p.R157*). While cerebrovascular manifestations such as MMD are rarely reported in patients with *NFKB1* haploinsufficiency, they have been observed in patients with CVID, often presenting as progressive stenosis or occlusion of intracranial vessels (20). Previous genetic studies have identified *RNF213* at 17q25.3 as a primary susceptibility gene for MMD, particularly in East Asian populations (21). However, no *RNF213* variants were detected in our patients, suggesting that the observed intracranial vasculitis may have been associated with the *NFKB1* haploinsufficiency.

In conclusion, our findings emphasize the importance of comprehensive genetic screening in such clinical presentations and suggest that targeted therapeutic strategies may be beneficial, particularly for those with severe or atypical features. A key limitation of our study is its relatively small size compared with larger cohort-based analysis. Further investigations into *NFKB1* haploinsufficiency are warranted to elucidate potential genotype-phenotype correlations and to develop additional screening approaches for assessing the lifetime risk of severe complications, especially through a deeper understanding of the underlying molecular mechanisms in affected tissues and immune cell subsets.

## Data Availability

The original contributions presented in the study are included in the article/**Supplementary Material**, further inquiries can be directed to the corresponding author/s.
