# Correction: Clinical and immunological characterization of NFKB1 haploinsufficiency in Japan

**DOI:** 10.3389/fimmu.2026.1933982

**Published:** 2026-08-10

**Authors:** Kunihiko Moriya, Yuji Kamiyama, Ryo Ogino, Shuya Kaneko, Takeshi Isoda, Takahiro Kamiya, Yuki Sakai, Fumi Hirose, Hidetoshi Hagiwara, Itaru Iwama, Ryusuke Nambu, Yoji Uejima, Fumiaki Sakura, Miyuki Tsumura, Kazushi Izawa, Yusuke Matsuda, Naoko Nakatani, Akihiro Tamura, Tomo Nozawa, Masaki Shimizu, Taizo Wada, Satoshi Okada, Hirokazu Kanegane, Kohsuke Imai

**Affiliations:** 1 Department of Pediatrics, National Defense Medical College, Saitama, Japan; 2 Department of Antibody Drug Development, Tohoku University Graduate School of Medicine, Sendai, Japan; 3 Department of Pediatrics, Graduate School of Medicine, Yokohama City University, Yokohama, Japan; 4 Department of Pediatrics, Kyoto University Graduate School of Medicine, Kyoto, Japan; 5 Department of Pediatrics and Developmental Biology, Graduate School of Medical and Dental Sciences, Institute of Science Tokyo, Tokyo, Japan; 6 Division of Gastroenterology and Hepatology, Saitama Children’s Medical Center, Saitama, Japan; 7 Department of Infectious Diseases and Immunology, Division of Infectious Diseases and Immunology, Saitama Children’s Medical Center, Saitama, Japan; 8 Department of Pediatrics, Hiroshima University Graduate School of Biomedical and Health Sciences, Hiroshima, Japan; 9 Department of Pediatrics, School of Medicine, Institute of Medical, Pharmaceutical and Health Sciences, Kanazawa University, Kanazawa, Japan; 10 Department of Pediatrics, Kobe University Graduate School of Medicine, Kobe, Japan; 11 Department of Child Health and Development, Graduate School of Medical and Dental Sciences, Institute of Science Tokyo, Tokyo, Japan

**Keywords:** autoimmunity, autoinfammatory disease, common variable immunodeficiency (CVID), inborn errors of immunity (IEI), NFKB1

There was a mistake in the caption of **Figure 1** and **Figure 3** as published. The captions for these figures were inadvertently switched. The corrected captions of **Figure 1** and **3** appear below.

“Figure 1. AD NFKB1 deficiency in 9 families. **(A)** Pedigree of the 9 unrelated families showing familial segregation of the different NFKB1 alleles. Male and female individuals are represented by squares and circles, respectively. Symptomatic individuals are represented by closed black symbols, and asymptomatic carriers are indicated by a black vertical line. **(B)** Schematic representation of the NFKB1 gene. The positions of the variants observed in the individuals are indicated by arrows. RHD, Rel homology domain; GRR, glycine-rich region, ARD, ankyrin repeat domain; DD, death domain.”

“Figure 3. AD *NFKB1* variants do not underlie type I interferonopathy. **(A)** Relative expression of six ISGs and **(B)** IFN scores of 11 healthy controls, 8 patients with AD *NFKB1* variants, and 3 patients with Aicardi–Goutières syndrome (AGS) were evaluated by qRT–PCR. The relative abundance of each transcript was normalized to the expression level of β-actin, and the results are shown relative to a single calibrator. The median of the relative quantification of the 6 ISGs was used to calculate the “IFN score” for each patient. IFN scores greater than +2 SD of the average of 11 healthy controls (5.04) were designated as positive. Two independent experiments were performed to confirm the results.”

The original version of this article has been updated.

