# Supplementary material for: Clinical and immunological characterization of NFKB1 haploinsufficiency in Japan
Source: Front Immunol. 2026 Jul 8;17:1866459. doi: 10.3389/fimmu.2026.1866459 (PMC13388040; doi:10.3389/fimmu.2026.1866459)
Supplement: Supplementary file 1 [file Table1.docx]

**Supplementary Table 1. Flowcytometric analysis of patients with AD NFKB1 deficiency**

|  | **A-1** | **A-2** | **B-1** | **B-2** | **C-1** | **D-1** |
| --- | --- | --- | --- | --- | --- | --- |
| **T cells** |  |  |  |  |  |  |
| T cells (% of Lymphocytes) | 25.1 % | 59.0 % | 30.6 % | 34.6 % | 91.6% | 78.0 % |
| Helper T cells (% of CD3+) | 36.5 % | 64.5 % | 34.14 % | 40.81 % | NT | 43.1 % |
| Cytotoxic T cells (% of CD3+) | 38.0 % | 30.1 % | 54.79 % | 49.64 % | NT | 48.1 % |
| CD4/8 ratio | 0.96 | 2.14 | 0.62 | 0.82 | NT | 0.90 |
| Naïve Th cells (% of CD3+CD4+) | 62.1 % | 54.2 % | 59.32 % | 84.65 % | NT | 50.3 % |
| Recent thymic emigrants  (% of CD3+CD4+ +CD45RA+) | 42.6 % | 30.2 % | 51.18 % | 79.9 % | NT | 22.3 % |
| CD4+ memory T cells  (% of CD3+CD4+CD45RO+) | 33.5 % | 38.7 % | 31.36 % | 9.21 % | NT | 53.5 % |
| CD8+ memory T cells  (% of CD3+CD8+CD45RO+) | 23.2 % | 23.7 % | 28.13 % | 19.41 % | NT | 27.2 % |
| γδ Tcells (% of CD3+) | 15.2 % | 0.68 % | 9.59 % | 17.76 % | NT | 7.25 % |
| Double negative Tcells  (% of CD3+TCRαβ+) | 0.35 % | 0.071 % | 0.00 % | 0.04 % | NT | 0.73 % |
| Th1 cells (% of CD3+CD4+CD45RO+) | 41.9 % | 21.2 % | 9.72 % | 2.74 % | NT | 0.45 % |
| Th2 cells (% of CD3+CD4+CD45RO+) | 40.6 % | 49.7 % | 67.08 % | 87.59 % | NT | 88.0 % |
| Th17 cells (% of CD3+CD4+CD45RO+) | 2.96 % | 8.56 % | 13.93 % | 4.52 % | NT | 4.30 % |
| Regulatory T cells  (% of CD3+CD4+CD25^hi^CD127^low^) | 9.16 % | 2.94 % | 4.55 % | 3.08 % | NT | 3.92 % |
| **NK cells** |  |  |  |  |  |  |
| CD56+CD16+ (% of lymphocytes) | 47.0 % | 32.0 % | 45.96 % | 13.55 % | NT | 10.7 % |
| **B cells** |  |  |  |  |  |  |
| B cells (% of lymphocytes) | 15.5 % | 0.18 % | 24.39 % | 45.33 % | 0% | 4.29 % |
| Naïve B cells (% of CD19+) | 68.7 % | 2.44 % | 67.65 % | 78.22 % | NT | 62.4 % |
| Memory B cells (% of CD19+) | 4.89 % | 17.6 % | 21.28 % | 11.8 % | NT | 19.2 % |
| Switched memory B cells (% of CD19+) | 7.69 % | 17.1 % | 5.36 % | 2.46 % | NT | 2.57 % |
| Merginal Zone B cells (% of CD19+) | 18.0 % | 9.76 % | 22.97 % | 15.91 % | NT | 31.5 % |
| CD38^High^IgM^High^ Transitional B cells  (% of CD19+) | 35.9 % | 11.5% | 3.58 % | 3.27 % | NT | 5.20 % |
| CD38^High^CD24^High^ Transitional B cells  (% of CD19+) | 30.0 % | 3.85 % | 1.25 % | 3.80 % | NT | 4.55 % |
| CD24++CD10+ Transitional B cells  (% of CD19+) | 35.7 % | 7.69 % | NT | NT | NT | 4.03 % |
| Plasmablasts (% of CD19+) | 2.07 % | 46.2 % | 2.56 % | 0.34 % | NT | 0.90 % |
| IgG+ memory B cells (% of CD19+) | 4.23 % | 7.32 % | 1.51 % | 0.34 % | NT | 0.33 % |
| IgA+ memory B cells (% of CD19+) | 2.03 % | 2.44 % | 4.26 % | 1.20 % | NT | 0.29 % |
| IgM+ B cells (% of CD19+) | 83.1 % | 24.4 % | 86.47 % | 89.35 % | NT | 78.5 % |
| IgD+ B cells (% of CD19+) | 85.5 % | 12.2 % | 89.30 % | 92.02 % | NT | 87.1 % |
| **Immunoglobulins (Ig)** |  |  |  |  |  |  |
| IgG (mg/dL) | 1003 | 67 | NT | 410 | 10 | 26 |
| IgA (mg/dL) | 13 | < 3 | NT | 38 | <3 | 1 |
| IgM (mg/dL) | 45 | < 2 | NT | 20 | <2 | 7 |

|  | **E-1** | **E-2** | **F-1** | **F-2** | **G-1** |
| --- | --- | --- | --- | --- | --- |
| **T cells** |  |  |  |  |  |
| T cells (% of Lymphocytes) | 52.1 % | 62.0 % | 62.1 % | 67.1 % | 81.1 % |
| Helper T cells (% of CD3+) | 25.6 % | 60.5 % | 83.2 % | 56.6 % | 62.8 % |
| Cytotoxic T cells (% of CD3+) | 69.4 % | 33.8 % | 14.1% | 38.3 % | 33.1 % |
| CD4/8 ratio | 0.39 | 1.79 | 5.90 | 1.48 | 1.90 |
| Naïve Th cells (% of CD3+CD4+) | 5.31 % | 42.5 % | 4.17 % | 37.4 % | 38.1 % |
| Recent thymic emigrants  (% of CD3+CD4+ +CD45RA+) | NT | 71.6 % | 64.9 % | 93.1 % | 38.5 % |
| CD4+ memory T cells  (% of CD3+CD4+CD45RO+) | 22.2 % | 20.0 % | 81.6 % | 23.2 % | 29.8 % |
| CD8+ memory T cells  (% of CD3+CD8+CD45RO+) | 27.3 % | 7.17 % | 9.12 % | 8.10 % | 14.0 % |
| γδ Tcells (% of CD3+) | NT | 4.55 % | 1.86 % | 3.10 % | 2.04 % |
| Double negative Tcells  (% of CD3+TCRαβ+) | NT | 0.39 % | 0.41 % | 0.98 % | 1.07 % |
| Th1 cells (% of CD3+CD4+CD45RO+) | NT | 43.1 % | 6.69 % | 37.8 % | 36.1 % |
| Th2 cells (% of CD3+CD4+CD45RO+) | NT | 21.7 % | 77.7 % | 25.7 % | 34.8 % |
| Th17 cells (% of CD3+CD4+CD45RO+) | NT | 9.93 % | 14.5 % | 12.1 % | 13.1 % |
| Regulatory T cells  (% of CD3+CD4+CD25^hi^CD127^low^) | NT | 3.20 % | 4.02 % | 5.34 % | 5.65 % |
| **NK cells** |  |  |  |  |  |
| CD56+CD16+ (% of lymphocytes) | 38.5 % | 15.8 % | 16.8 % | 12.0 % | 9.20 % |
| **B cells** |  |  |  |  |  |
| B cells (% of lymphocytes) | 0.073 % | 13.9 % | 3.13 % | 13.9 % | 3.58 % |
| Naïve B cells (% of CD19+) | NT | 89.9 % | 82.0 % | 87.9 % | 84.7 % |
| Memory B cells (% of CD19+) | 11.9 % | 8.03 % | 5.04 % | 8.20 % | 11.8 % |
| Switched memory B cells (% of CD19+) | NT | 3.54 % | 1.01 % | 5.42 % | 6.42 % |
| Merginal Zone B cells (% of CD19+) | NT | 4.26 % | 4.38 % | 5.07 % | 4.78 % |
| CD38^High^IgM^High^ Transitional B cells  (% of CD19+) | NT | NT | NT | NT | NT |
| CD38^High^CD24^High^ Transitional B cells  (% of CD19+) | NT | 5.48 % | 0.00 % | 5.32 % | 3.51 % |
| CD24++CD10+ Transitional B cells  (% of CD19+) | NT | 7.76 % | 0.17 % | 3.98 % | 3.32 % |
| Plasmablasts (% of CD19+) | NT | 0.62 % | 0.17 % | 3.34 % | 1.64 % |
| IgG+ memory B cells (% of CD19+) | NT | NT | NT | NT | NT |
| IgA+ memory B cells (% of CD19+) | NT | NT | NT | NT | NT |
| IgM+ B cells (% of CD19+) | NT | NT | NT | NT | NT |
| IgD+ B cells (% of CD19+) | NT | NT | NT | NT | NT |
| **Immunoglobulins (Ig)** |  |  |  |  |  |
| IgG (g/L) | 115 | 1192 | 285 | 1278 | NT |
| IgA (g/L) | <10 | 101 | 23 | 140 | NT |
| IgM (g/L) | 8 | 80 | 13 | 57 | NT |

|  | **H-1** | **H-2** | **H-3** | **H-4** | **H-5** | **H-6** |
| --- | --- | --- | --- | --- | --- | --- |
| **T cells** |  |  |  |  |  |  |
| T cells (% of Lymphocytes) | 51.8 % | 19.8 % | 27.6 % | 71.1 % | 75.0 % | 75.1 % |
| Helper T cells (% of CD3+) | 53.6 % | 46.2 % | 38.4 % | 35.3 % | 45.4 % | 39.1 % |
| Cytotoxic T cells (% of CD3+) | 37.7 % | 45.8 % | 47.5 % | 48.4 % | 44.7 % | 52.8 % |
| CD4/8 ratio | 1.42 | 1.00 | 0.80 | 0.73 | 1.02 | 0.74 |
| Naïve Th cells (% of CD3+CD4+) | 43.9 % | 70.6 % | 64.2 % | 43.7 % | 82.1 % | 81.4 % |
| Recent thymic emigrants  (% of CD3+CD4+ +CD45RA+) | 35.6 % | 47.2 % | 41.9 % | 39.8 % | 63.1 % | 70.4 % |
| CD4+ memory T cells  (% of CD3+CD4+CD45RO+) | 48.3 % | 26.2 % | 33.9 % | 54.5 % | 18.4 % | 17.3 % |
| CD8+ memory T cells  (% of CD3+CD8+CD45RO+) | 32.0 % | 7.97 % | 6.93 % | 9.76 % | 3.99 % | 4.37 % |
| γδ Tcells (% of CD3+) | 1.09 % | 6.20 % | 14.0 % | 10.5 % | 84.5 % | 7.95 % |
| Double negative Tcells  (% of CD3+TCRαβ+) | 0.20 % | 0.49 % | 0.43 % | 0.86 % | 8.04 % | 1.05 % |
| Th1 cells (% of CD3+CD4+CD45RO+) | 21.0 % | 10.2 % | 17.2 % | 27.9 % | 0.74 % | 1.52 % |
| Th2 cells (% of CD3+CD4+CD45RO+) | 60.7 % | 74.8 % | 69.3 % | 54.3 % | 1.51 % | 90.7 % |
| Th17 cells (% of CD3+CD4+CD45RO+) | 6.73 % | 2.87 % | 2.74 % | 6.77 % | 89.3 % | 1.98 % |
| Regulatory T cells  (% of CD3+CD4+CD25^hi^CD127^low^) | 4.98 % | 3.55 % | 3.19 % | 2.04 % | 3.70 % | 3.29 % |
| **NK cells** |  |  |  |  |  |  |
| CD56+CD16+ (% of lymphocytes) | 13.3 % | 0.76 % | 2.63 % | 2.56 % | 4.69 % | 1.97 % |
| **B cells** |  |  |  |  |  |  |
| B cells (% of lymphocytes) | 20.8 % | 9.47 % | 17.0 % | 17.5 % | 15.7 % | 18.3 % |
| Naïve B cells (% of CD19+) | 69.7 % | 8.82 % | 6.66 % | 72.1 % | 91.0 % | 86.0 % |
| Memory B cells (% of CD19+) | 14.2 % | 84.1 % | 82.5 % | 5.5 % | 3.84 % | 6.02 % |
| Switched memory B cells (% of CD19+) | 12.6 % | 5.44 % | 6.12 % | 4.15 % | 2.00 % | 4.18 % |
| Merginal Zone B cells (% of CD19+) | 15.5 % | 6.39 % | 4.00 % | 20.6 % | 4.74 % | 6.07 % |
| CD38^High^IgM^High^ Transitional B cells  (% of CD19+) | 3.10 % | 5.90 % | 1.37 % | 0.01% | 7.15 % | 6.78 % |
| CD38^High^CD24^High^ Transitional B cells  (% of CD19+) | 0.49 % | 5.05 % | 0.77 % | 0.00% | 7.15 % | 8.98 % |
| CD24++CD10+ Transitional B cells  (% of CD19+) | 5.30 % | 4.70 % | 1.92 % | 1.02 % | 6.84 % | 11.4 % |
| Plasmablasts (% of CD19+) | 0.18 % | 1.42 % | 2.30 % | 0.30 % | 0.077 % | 0.22 % |
| IgG+ memory B cells (% of CD19+) | 0.28 % | 0.056 % | 0.14 % | 0.24 % | 0.20 % | 0.076 % |
| IgA+ memory B cells (% of CD19+) | 2.04 % | 1.01 % | 1.10 % | 0.95 % | 0.33 % | 0.81 % |
| IgM+ B cells (% of CD19+) | 79.2 % | 57.6 % | 66.9 % | 89.9 % | 52.8 % | 40.5 % |
| IgD+ B cells (% of CD19+) | 84.0 % | 84.8 % | 80.1 % | 91.6 % | 91.9 % | 86.5 % |
| **Immunoglobulins (Ig)** |  |  |  |  |  |  |
| IgG (g/L) | NT | 929 | 1059 | 269 | NT | NT |
| IgA (g/L) | NT | 130 | 146 | 30 | NT | NT |
| IgM (g/L) | NT | 31 | 79 | 24 | NT | NT |

（

|  | **I-2** | **I-3** |
| --- | --- | --- |
| **T cells** |  |  |
| T cells (% of Lymphocytes) | 84.9 % | 51.4 % |
| Helper T cells (% of CD3+) | 57.1 % | 38.4 % |
| Cytotoxic T cells (% of CD3+) | 32.3 % | 34.5 % |
| CD4/8 ratio | 1.77 | 1.11 |
| Naïve Th cells (% of CD3+CD4+) | 61.3 % | 69.5 % |
| Recent thymic emigrants  (% of CD3+CD4+ +CD45RA+) | 47.7 % | 56.1 % |
| CD4+ memory T cells  (% of CD3+CD4+CD45RO+) | 24.5 % | 17.7 % |
| CD8+ memory T cells  (% of CD3+CD8+CD45RO+) | 10.5 % | 7.13 % |
| αβ Tcells (% of CD3+) | 85.5 % | 72.6 % |
| γδ Tcells (% of CD3+) | 11.6 % | 24.9 % |
| Double negative Tcells  (% of CD3+TCRαβ+) | NT | NT |
| Th1 cells (% of CD3+CD4+CD45RO+) | 1.79 % | 2.89 % |
| Th2 cells (% of CD3+CD4+CD45RO+) | 94.5 % | 93.2 % |
| Th17 cells (% of CD3+CD4+CD45RO+) | 0.82 % | 0.95 % |
| Regulatory T cells  (% of CD3+CD4+CD25^hi^CD127^low^) | 0.94 % | 0.71 % |
| **NK cells** |  |  |
| CD56+CD16+ (% of lymphocytes) | 8.12 % | 12.0 % |
| **B cells** |  |  |
| B cells (% of lymphocytes) | 0.35 % | 23.5 % |
| Naïve B cells (% of CD19+) | 57.0 % | 87.4 % |
| Memory B cells (% of CD19+) | 2.44 % | 4.21 % |
| Switched memory B cells (% of CD19+) | 15.7 % | 2.54 % |
| Merginal Zone B cells (% of CD19+) | 8.92 % | 5.03 % |
| CD38^High^IgM^High^ Transitional B cells  (% of CD19+) | 63.9 % | 12.0 % |
| CD38^High^CD24^High^ Transitional B cells  (% of CD19+) | 70.1 % | 10.1 % |
| CD24++CD10+ Transitional B cells  (% of CD19+) | 72.2 % | 8.66 % |
| Plasmablasts (% of CD19+) | 0.83 % | 0.063 % |
| IgG+ memory B cells (% of CD19+) | 2.89 % | 0.16 % |
| IgA+ memory B cells (% of CD19+) | 1.31 % | 0.41 % |
| IgM+ B cells (% of CD19+) | 64.6 % | 74.0 % |
| IgD+ B cells (% of CD19+) | 61.7 % | 83.9 % |
| **Immunoglobulins (Ig)** |  |  |
| IgG (g/L) | <6 | 303 |
| IgA (g/L) | <4 | 4 |
| IgM (g/L) | <2 | 22 |
